# Measurement of aerodynamic force and moment acting on a javelin using a magnetic suspension and balance system

**DOI:** 10.1038/s41598-023-27534-2

**Published:** 2023-01-09

**Authors:** Kazuya Seo, Hiroyuki Okuizumi, Yasufumi Konishi, Takuto Kobayashi, Hiroaki Hasegawa, Shigeru Obayashi

**Affiliations:** 1 Department of Mechanical Engineering, Kogakuin University, Tokyo, 1618677 Japan; 2 Institute of Fluid Science, Tohoku University, Sendai, 9808577 Japan; 3 Department of Science, Yamagata University, Yamagata, 9908560 Japan; 4 Department of Mechanical System Engineering, Utsunomiya University, Utsunomiya, 3218585 Japan

**Keywords:** Aerospace engineering, Mechanical engineering

## Abstract

The rules governing the dimensions of the Javelin were substantially changed in 1986. It was considered that this new design guaranteed there was zero pitching moment at 0° angle of attack and that the pitching moment decreased (became negative) with increasing angle of attack. The objective of this study is to investigate if the pitching moment remains always negative (nose-down rotation). To measure accurate aerodynamic forces acting on a Javelin, the world’s largest 1 m magnetic suspension and balance system was used. The magnetic suspension and balance system was able to measure aerodynamic forces without support interference in the wind tunnel. In addition, computational fluid dynamics were carried out to estimate the pitching moment coefficients. It was found that the pitching moment coefficient of a commercially available Javelin becomes positive (nose-up rotation) at lower angles of attack, less than 12°. The pitching moment becomes positive if the upstream side of the center of gravity receives more inflow than the downstream side. This situation can be attained by, for example, increasing the thickness of the upstream side when compared with that of the downstream side.

## Introduction

A conventional wind tunnel test for a Javelin would be performed by fixing the Javelin to a supporting rod^1,2^. However, the supporting rod disturbs the flow and this is known as support interference. For example, the separation line on the ellipsoid has been observed to drastically move backwards^3^ when using a thin rod whose diameter is 0.5 mm. Generally, in the case of the equipment of sports, the sizes are comparable with hands or feet. In particular, for a long and narrow object like the javelin, support interference becomes significant because the diameter of the supporting rod is comparable to that of the javelin, making it difficult to measure the aerodynamic forces accurately^4^.

A Magnetic Suspension and Balance System (MSBS) is a valuable tool for measuring aerodynamic forces without support interference. The first MSBS was developed in ONERA in 1950s^5^. However, further research and development of this MSBS has been suspended since 1970s. This was because there was no prospect of commercialization of MSBS^6^. Nowadays, research and development of MSBS has been restarted in ODU^7^, KAIST^8^ and Tohoku University^9^ due to improved sophistication of the measuring equipment and improved computer control, as well as the development of powerful neodymium magnets. However, there are still relatively few MSBS’s in the world.

From the view of aerodynamics of the Javelin, the rules governing the dimensions were substantially changed in 1986. The main factor that motivated the change was that in many throws the Javelin was landing nearly flat, causing difficulties for the judges to determine whether the throw was valid or not^10^. It was considered that the new design guaranteed that the pitching-moment profile of the Javelin was monotonically decreasing with increasing the angle of attack, without ever attaining a positive value.

The objective of this study is to make sure whether the pitching moment is always negative (nose-down rotation) with respect to the angle of attack. The aerodynamic forces acting on the Javelin without the supporting rod will be described. The world’s largest magnetic suspension and balance system (MSBS) was employed to measure the aerodynamic forces acting on a full-size women’s Javelin. Therefore, the aerodynamic coefficients shown in this paper should be the most accurate. In addition, a parametric study using CFD (Computational Fluid dynamics) was also carried out assess whether the pitching moment is always negative.

## Methods

### Principle of the magnetic suspension and balance system

**Figure 1:**
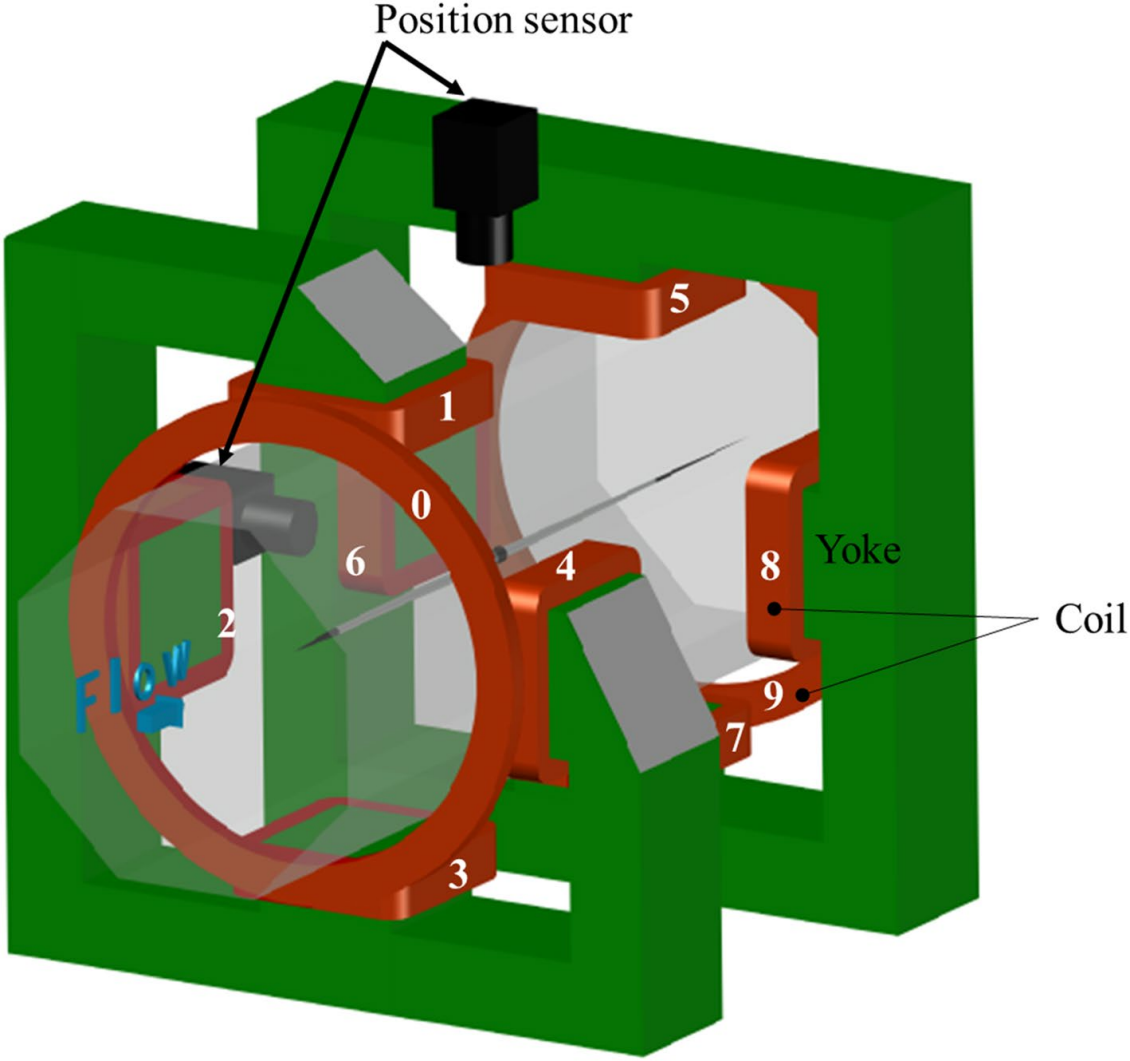
A schematic of the magnetic suspension and balance system.

The magnetic suspension and balance system (MSBS) is shown in Fig. 1. A Javelin which includes magnets along the longitudinal axis is levitated at the center of the test section. When wind flows, aerodynamic forces act on the Javelin and the control principle is designed to keep the Javelin at the center of the test section (home position). To keep the same position and the same attitude of the Javelin, ten coils are placed around the test section. For example, the two donut-like air core coils (#0 and #9) in the flow direction work to counterbalance the drag. The other eight coils are iron core coils, which efficiently generate a magnetic field by connecting coils # 1 to # 4 and # 5 to # 8 with the yoke to form a magnetic circuit^11^. A power amplifier is attached to each coil, each of which can pass a current of up to 150 A. The current of the coils is adjusted to keep the same position and the same attitude. The drive current differences between the wind-on condition and the wind-off condition are converted into aerodynamic forces. In the case of a vibrating javelin, the measured currents with the wind-on condition include both components of aerodynamic forces and inertial forces. On the other hand, the measured currents with the wind-off condition of the vibrating javelin include only inertial forces. Therefore, the differences in current between the wind-on condition and the wind-off condition can be converted into aerodynamic forces and moments.

### Javelin

**Figure 2:**
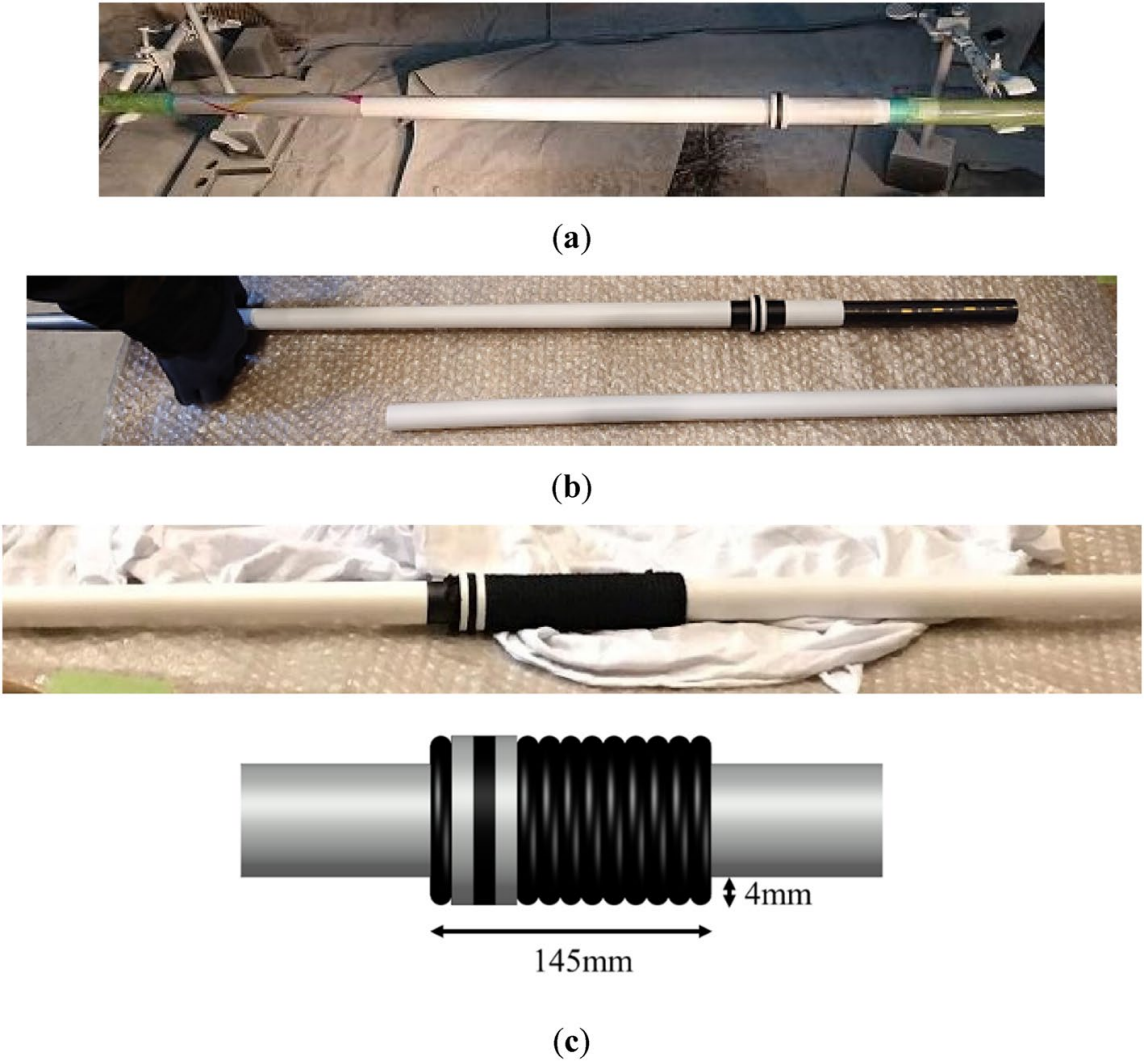
Commercially available women’s javelin: (**a**) The javelin was spray painted white and a 15 mm-long collar was attached to the javelin at the center of gravity; (**b**) Neodymium magnets were inserted along the longitudinal axis. (**c**) A 15 mm-long-collar and grip string.

A commercially available full-size women’s Javelin (Hybrid Genome X, Nishi) was employed for the wind tunnel tests^12^. The length of the Javelin was 2210 mm, and the center of gravity was at 920 mm from the tip. Most of the surface of the Javelin was spray painted white, as shown in Fig. 2a, to aid detecting the position. For the same reason, a 15-mm-long collar was attached at the center of gravity, and 5-mm-wide black tape was wrapped around the Javelin at the center of gravity. Neodymium magnets were inserted along the longitudinal axis, as shown in Fig. 2b. Two types of the magnet with diameters of 19 mm and 20 mm were used, and the total length of the magnet assembly was 495 mm. Grip strings were wound around the javelin both on the upstream and downstream sides of the collar as shown in Fig. 2c. The diameter of the strings, which is 4 mm, has the same width as the height of the 15-mm-long collar.

### Position-sensing system

**Figure 3:**
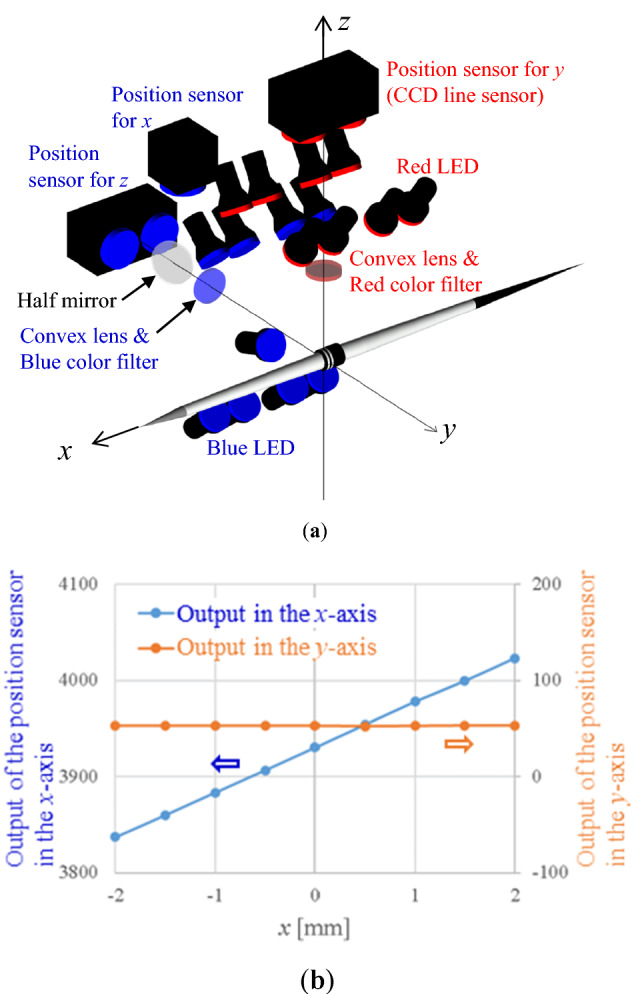
The position-sensing system: (**a**) Schematic of the position-sensing system; (**b**) An example of the calibration results of position sensor in the *x*-axis.

A schematic of the optical position-sensing system is shown in Fig. 3a. The coordinate system is also shown. The origin was at the center of gravity of the javelin, with the positive *x*-axis in the horizontal upstream direction, the *y*-axis was also horizontal and orthogonal to the *x*-axis. The positive *z*-axis was vertically upward. The optical position-sensing system is composed of a convex lens (focal length 125 mm), dichroic color filters (red and blue), a half mirror, red and blue LED lights (MSPP-CB74, Moritex), and position sensors which are CCD (Charge-Coupled Device) line sensor camera (TL7450S, Takenaka system equipment). CCD line sensor camera is composed of 7450 CCDs in a line. The size of the CCD element is 4.7 μm times 4.7 μm, and the pixel resolution is less than 10 μm. The sampling frequency is 1250 Hz.

### Position calibration

The real position and attitude were defined by the five-component stages (ALS-904H1P, ALV-104HP, ATS-130HP & ARS-936HP, Central Motor Wheel). The position sensors were calibrated with the defined position and attitude. An example of the calibration results in the *x*-axis are shown in Fig. 3b. In this case, the five-component stages were moved only in the *x*-axis. The output value from the position sensor on the *x*-axis varies linearly with respect to the real position change in the *x*-axis. Since the five-component stages moved in the only *x*-axis, the output counting value in the *y*-axis did not change (insensitive in the *y*-axis). Other axes were calibrated in the same manner.

### Levitation of the javelin and use of a notch filter

**Figure 4:**
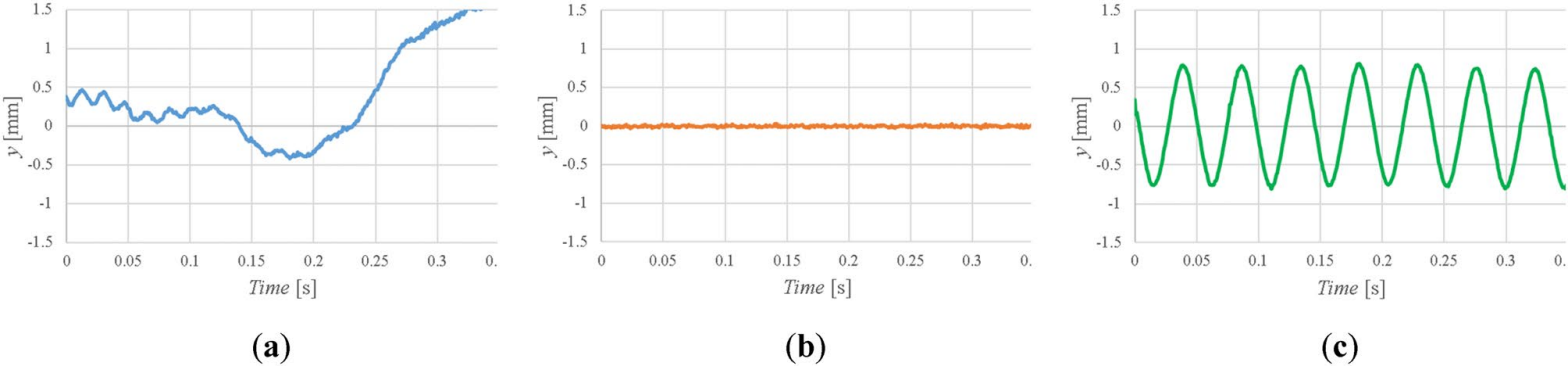
Time variations of the center of gravity of the javelin in the y-direction: (**a**) without the notch filter; (**b**) with the notch filter; (**c**) with a weak notch filter.

The first trial to levitate the Javelin failed. The time variations in the *y*-direction are shown in Fig. 4a. The javelin was unstable and diverged from its initial position after only 0.25 s^12^. The frequency components observed were 22 Hz and 55 Hz. The frequency of 22 Hz corresponded to the principle resonant frequency of the javelin^13,14^ and was the primary reason we were unable to control it. Therefore, a notch filter (band-stop filter) was employed to cut out the resonant frequency. As can be seen in Fig. 4b, the notch filter stabilized the Javelin, allowing us to levitate the javelin in the MSBS.

**Figure 5:**
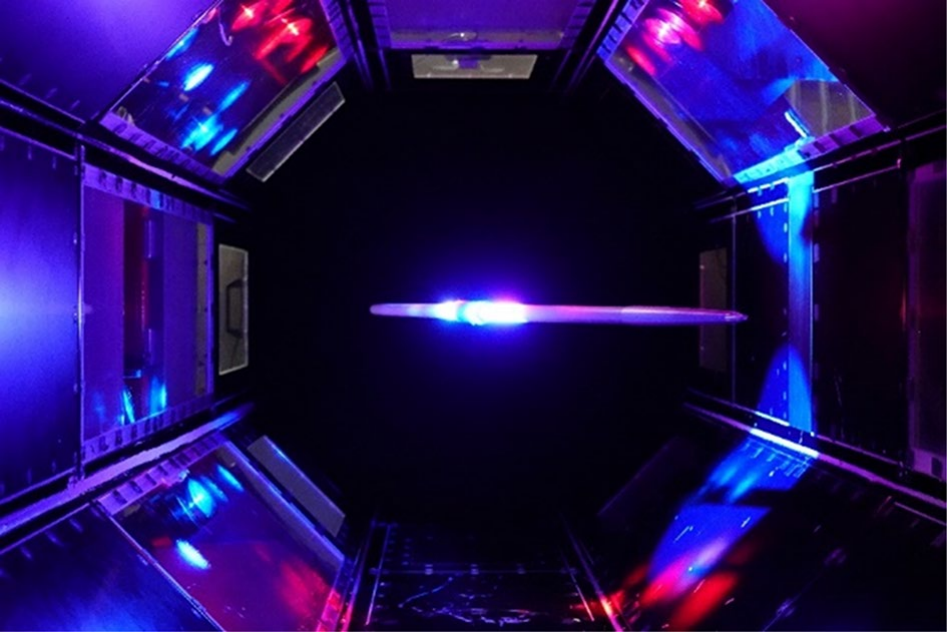
Javelin at 18° in the magnetic suspension and balance system.

In principle, the javelin should be always stabilized at the same position and the same attitude in the MSBS. However, the presence of the resonance enabled us to realize a vibrating model in the MSBS with the resonance frequency of the Javelin. Figure 4c shows the time variation of *y*-direction with a weak notch filter, i.e., a filter with decreased intensity. By decreasing the intensity of the notch filter, vibration of the Javelin, as observed in real flight, was realized. The frequency of the vibration was 22 Hz, as before, but the Javelin remained under control. Figure 5 shows the javelin levitated in the test section in the wind tunnel. It was illuminated brightly around the center of gravity to detect the position. The *AoA* is 18°, which is the largest value we can use in the world's largest MSBS. This is because the tail of the javelin is approaching the wall of the test section, but still outside of the boundary layer of the wall at 18° and because the LED lighting to detect the position can’t illuminate the Javelin at more than 18°. The *AoA* was changed in the horizontal plane (on the vertical *z*-axis). This definition of *AoA* allowed us to decrease the current when compared with the change of *AoA* in the vertical plane.

### Control system

**Figure 6:**
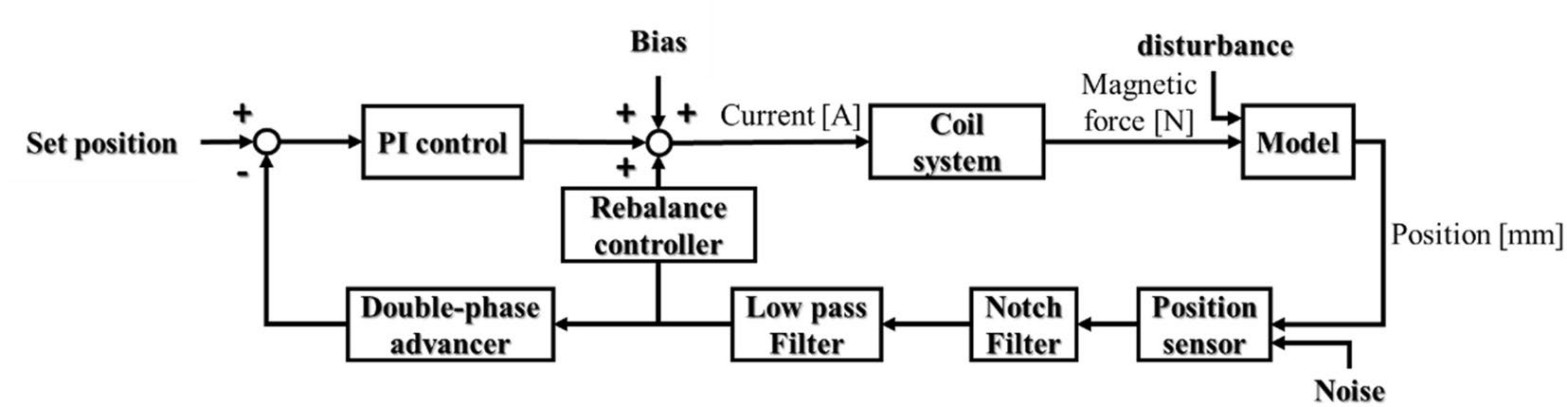
Block diagram of the control system.

The current in each coil is controlled by a proportional-integral (PI) controller and a double phase advancer^15^, as shown in Fig. 6. The PI controller reduces the deviation between the detected position and the set position, while the double phase advancer is used to compensate for the time delay of the signal from the position sensor passed through the two filters. To enable the constants of the proportional-integral controller and the double phase advancer to be determined, a square-wave signal (step waveform) is input to the coil system, and the constants are evaluated from the proximity of the results to those from a model of it.

### Force calibration

**Figure 7:**
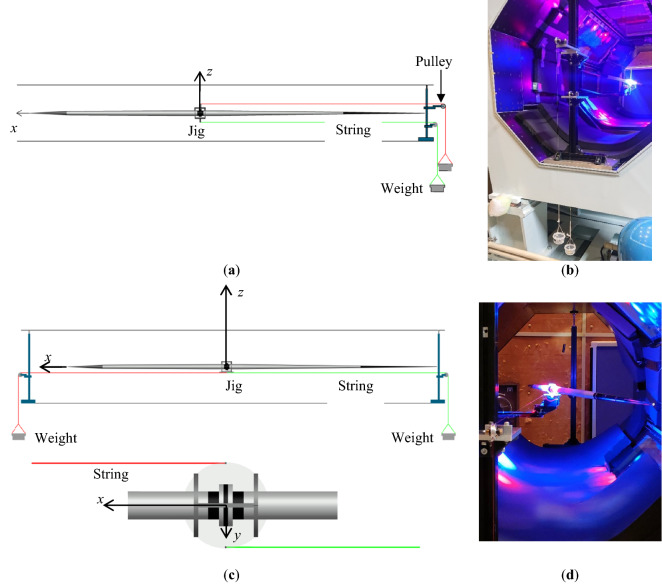
Force and Moment calibration: (**a**) Schematic of the force calibration in the x-direction; (**b**) Picture of the force calibration in the x-direction from the downstream side; (**c**) Schematic of the moment calibration in the z-direction; (**d**) Picture of the moment calibration on the z-axis with *AoA* of 16° from the downstream side.

To relate the forces and the moment to the current, several weights were applied as calibration references. For example, Fig. 7a and b show the force calibration in the *x*-direction. Figure 7a is a schematic, while Fig. 7b shows a picture from the downstream side. Two cups and two light strings were used to apply weights via a jig and pulleys in the *x*-direction only. The weights, *F*_*x*_, were applied to the levitated javelin. The force in the *y*-axis was calibrated in the same manner. The moment on the z-axis was also calibrated as shown in Fig. 7c and d. A 50 mm diameter disk was attached just below the center of gravity, and two weights were applied to the disk via pulleys. A weight was attached via strings to each side of the disk, applying forces on the upstream (+ *x* direction) and downstream (− *x* direction) sides. It can be seen from Fig. 7d that one string is attached to one side of the disk, and is pulled downstream by the weight via a pulley and that the other string is attached to the other side of the disk, and is pulled upstream by the weight via a pulley. In this way, a moment, *N*_*z*_, was applied to the levitated javelin.

**Figure 8:**
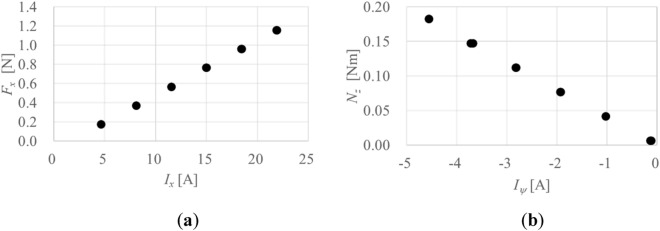
Calibration results: (**a**) *F*_*x*_; (**b**) *N*_*z*_.

The calibration results of *F*_*x*_ and *N*_*z*_ are shown in Fig. 8a and b, respectively. The absolute values of the applied currents increase linearly with increasing *F*_*x*_ and *N*_*z*_. The forces and the moment acting on the javelin in the wind-on condition can be calculated on the basis of these linear relationships.

### Wind tunnel

The low turbulence wind tunnel facility at the Institute of fluid science, Tohoku university was used for our work^16^. Since the distance between opposite sides of the hexagonal bell-mouth is 1.01 m, it was possible to employ a full-size women’s javelin in a wide range of angle of attack (*AoA*) up to 18°. The wind tunnel turbulence levels are among the lowest (less than 0.02% at 25 m/s) in the world. Moreover, the uniformity of its velocity profiles is within ± 0.02% with respect to the average velocity, making it possible to conduct very high-quality aerodynamics research. The experimental results presented in this paper would be expected to be very accurate because of the use of the MSBS without supporting interference and using such a large and low turbulence wind tunnel.

### Computational fluid dynamics (CFD)

The simulation was carried out using ANSYS 2021 R1, Design Modeler, Meshing and Fluent. A commercially available full-size women’s javelin (Hybrid Genome X, Nishi) was drawn using Design modeler. The length is 2.21 m, whilst the maximum diameter is 0.0247 m. The dimensions of the computational domain (enclosure) are 600 m × 600 m × 10 m in the vertical and lateral directions respectively.

**Figure 9:**
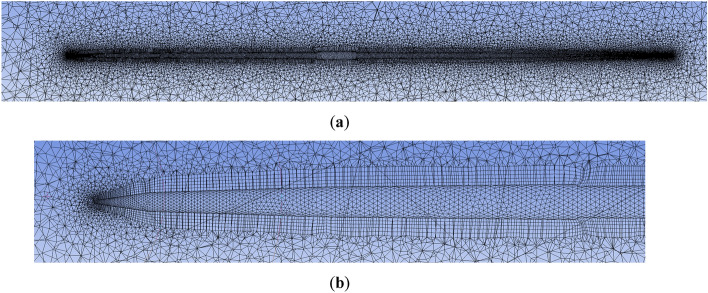
Meshes: (**a**) around the javelin; (**b**) around the javelin’s top.

Meshing was used for the computational domain. The summary is shown in Table 1. A first layer thickness inflation option was adopted to create an inflation mesh structure. The maximum skewness is about 0.89. Figure 9 shows meshes around the javelin (Fig. 9a) and the javelin’s top (Fig. 9b). Fluent was used to solve the 3D Reynolds-Average Navier–Stokes (RANS) equations and the continuity equation, using the finite volume method. A summary is also shown in Table 1. A standard k-ε model with standard wall functions is used for turbulence modeling.

**Table 1:** Summary of meshing and boundary conditions.

Set-up	Variable	Settings
Mesh	Element size	150 [mm]
	Nodes	3,703,025
	Elements	20,329,462
	Max skewness	0.89
Inflation	First layer thickness	2.0 [mm]
Turbulent model		Standard k-ε
Boundary condition	In: Velocity-inlet	25 [m/s]
	Out: Pressure-outlet	Gauge pressure:0 [Pa]
	Wall, Surface of javelin	Non-slip

## Results

### Experimental results of aerodynamic forces acting on a javelin

**Figure 10:**
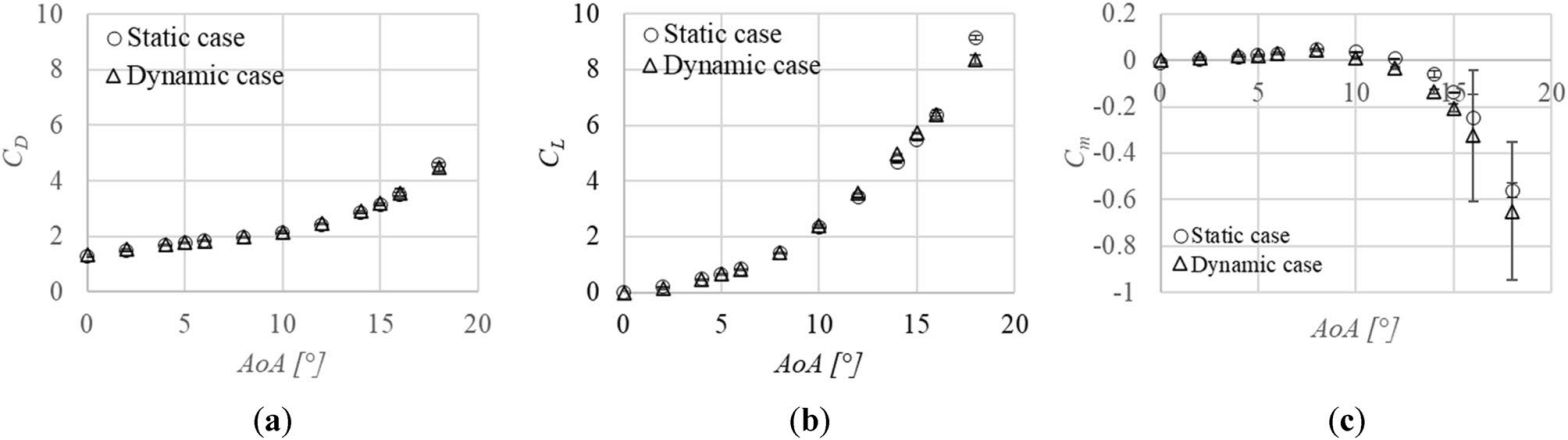
Aerodynamic coefficients as a function of the angle of attack, *AoA*. The static data are shown by open circles, while the dynamic (vibration) data are shown by open triangles. The 95% confidence intervals are also shown as error bars: (**a**) Drag coefficient, *C*_*D*_; (**b**) Drag coefficient, *C*_*L*_; (**c**) Pitching moment coefficient, *C*_*m*_.

The time averaged aerodynamic coefficients, *C*_*D*_, *C*_*L*_ and *C*_*m*_ on the static and the dynamic javelin are shown as a function of the angle of attack, *AoA* in Fig. 10. The *AoA* of the dynamic javelin is also the time averaged value, which is defined at 150 mm each on the upstream and downstream sides of the center of gravity. The 95% confidence intervals are also shown as error bars. The *C*_*D*_ and *C*_*L*_ are defined by Eqs. (1) and (2), while the *C*_*m*_ is defined by Eq. (3).

1 $$C_{D} = \frac{D}{{0.5\rho U^{2} A}}$$

2 $$C_{L} = \frac{L}{{0.5\rho U^{2} A}}$$

3 $$C_{m} = \frac{M}{{0.5\rho U^{2} Al}}$$

Here, *ρ* is the air density, *U* is the wind speed, *A* is the cross-sectional area at the maximum diameter (0.0247 mm), *l* is the length of the javelin (2.21 m).

The *C*_*D*_ and *C*_*L*_ for both cases increase with increasing *AoA* in the range between 0 and 18°. The error bars are small, that is, the measured data are very repeatable. The *C*_*m*_ becomes positive up to 10°. The *C*_*m*_ for both cases increases until 8°, then it decreases above that angle. The error bars are smaller at lower *AoA*. The value becomes negative above 12° and the absolute value increases with increasing *AoA*. When it becomes negative, the error bar becomes large and the date less repeatable. Longitudinal static stability concerned with the pitching moment occurs around about 12°. The *C*_*L*_ and *C*_*m*_ are almost 0 at 0°. The aerodynamic coefficients of the dynamic javelin are almost comparable with those of the static javelin.

**Figure 11:**
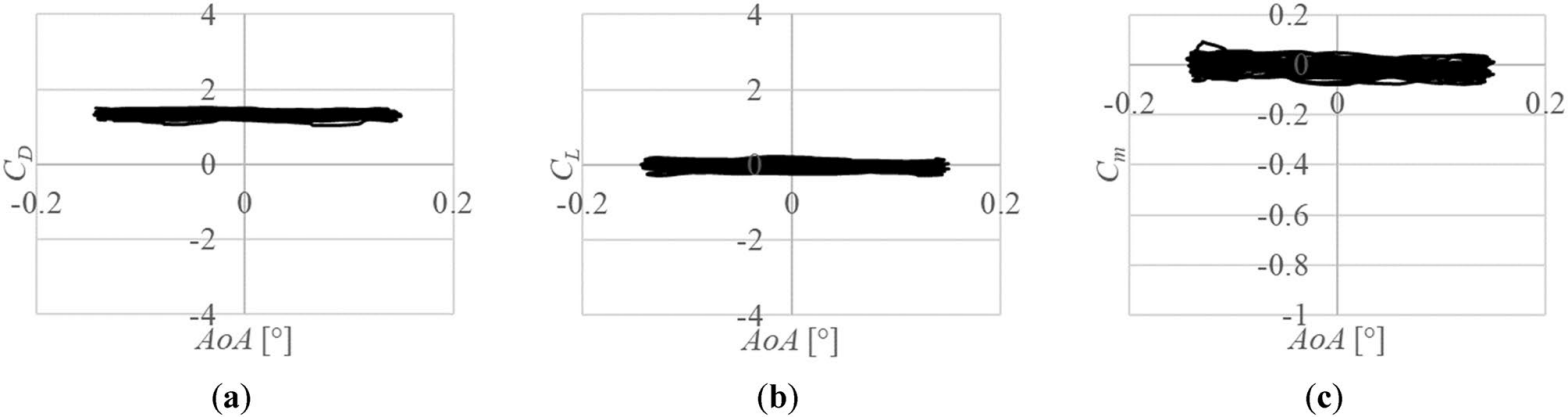
Aerodynamic coefficients of a vibrating javelin at the time-averaged *AoA* of 0° for one second: (**a**) Drag coefficient, *C*_*D*_; (**b**) Lift coefficient, *C*_*L*_; (**c**) Pitching moment coefficient, *C*_*m*_.

Figure 11 shows the aerodynamic coefficients, *C*_*D*_, *C*_*L*_ and *C*_*m*_, of a vibrating javelin for one second. Although the time-averaged *AoA* is 0°, it oscillates around 0° with an amplitude of 0.16°. The aerodynamic coefficients also oscillate around the time-averaged values, which coincide with the values of the dynamic case in Fig. 10. However, the amplitudes of the aerodynamic coefficients are very small, that is, they are almost constant even if *AoA* increases or decreases.

### CFD results

**Figure 12:**
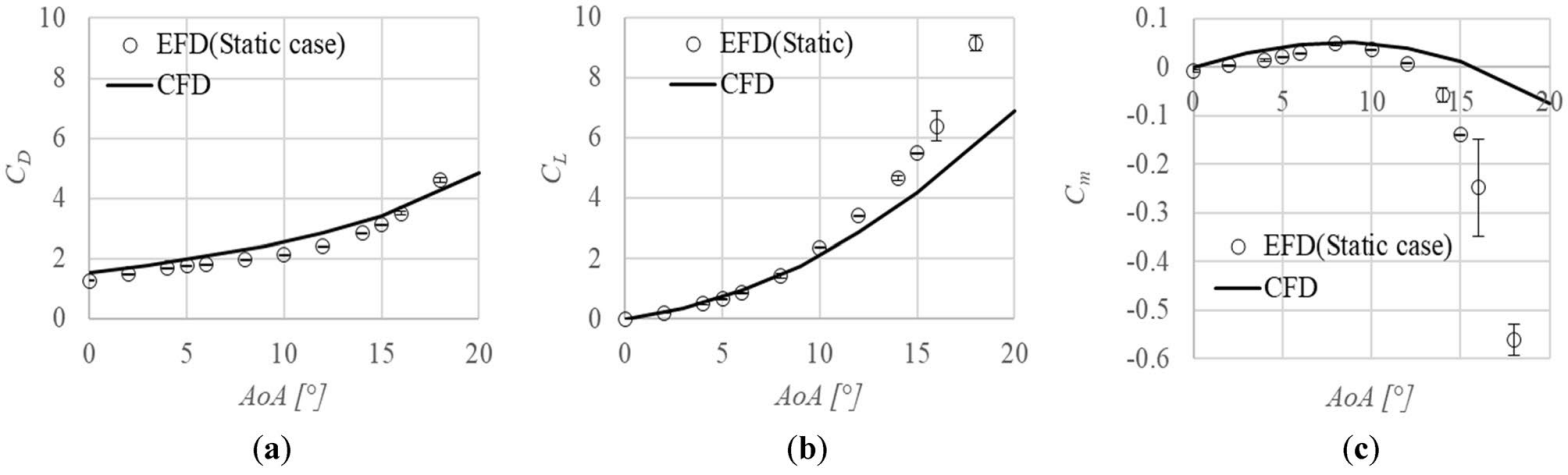
EFD (Experimental Fluid dynamics) results by MSBS are shown by open circles, while the CFD results are shown by the solid line as a function of the angle of attack, *AoA*. The 95% confidence intervals are also shown as error bars: (**a**) Drag coefficient, *C*_*D*_; (**b**) Drag coefficient, *C*_*L*_; (**c**) Pitching moment coefficient, *C*_*m*_.

The aerodynamic coefficients acting on the static javelin are shown in Fig. 12. The experimental fluid dynamics results (EFD (Static case)) are measured from the MSBS, while the computational fluid dynamics (CFD) results are calculated by Ansys Fluent. The EFD results in Fig. 10 are shown again. The *C*_*D*_ data obtained by CFD quantitatively agree with those by EFD, while the *C*_*L*_ and *C*_*m*_ obtained by CFD qualitatively agree with those by EFD. Therefore, the CFD can effectively simulate the aerodynamic coefficients on the javelin.

**Figure 13:**
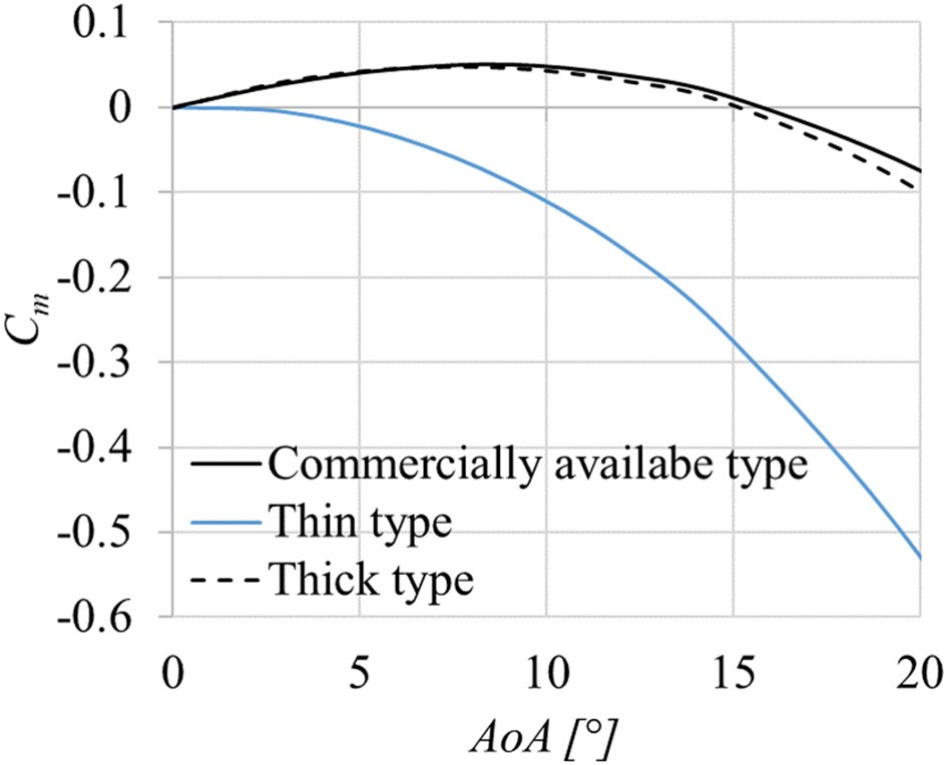
The pitching moment coefficient, *C*_*m*_, obtained by CFD for the three cases.

The *C*_*m*_ on the static javelin obtained by CFD for three cases are shown in Fig. 13. The difference amongst these three is the thickness of the top of the javelin. The thickness was changed within the range of the rules governing the dimensions of the javelin. It was found that the modern javelin still has positive values of *C*_*m*_ at lower *AoA*. In the case of the thinner top, the pitching-moment profile was monotonically decreasing with increasing *AoA*, without ever attaining a positive value as described in reference^10^.

## Discussion

It was found that the modern Javelin has positive values of *C*_*m*_ (nose-up) at lower *AoA,* as seen in Fig. 10c and Fig. 13. Consideration of whether the pitching moment is positive or not depends on the force difference between the upstream side and the downstream side of the center of gravity. If the force acting on the upstream side is larger than that on the downstream side at a certain *AoA*, a positive pitching moment around the center of gravity acts on the Javelin. Since the center of gravity of the Javelin is located at the upstream side from the geometric center, the pitching moment, in principle, tends to be negative. However, the pitching moment becomes positive, if the upstream side of the center of gravity gets more inflow than the downstream side. This situation can be attained by, for example, increasing the thickness on the upstream side when compared with that of the downstream side as shown in Fig. 13. The key to attain longer flight distances for ballistic motion is to decrease the drag in the first half and to increase the lift in the second half of the flight^17,18^. These are attained with a pitching-moment profile like Fig. 10c.

It has been reported that the vibrations of the javelin increase both lift and drag^13^. We observe that the aerodynamic coefficients of the dynamic (vibrating) javelin are almost comparable with those of the static Javelin, as shown in Fig. 10. Figure 10a shows that the *C*_*D*_ of the dynamic case is slightly larger than that of the static case at lower *AoA* as predicted by previous simulations^13^, but the difference is not as large as predicted in the previous simulations. On the other hand, Fig. 10b shows that the *C*_*L*_ of the dynamic case is slightly smaller than that of the static case. This is an opposite effect of the vibration to that predicted in reference^13^. This inconsistency between the previous simulation and the present data may occur because of the model assumed in reference^13^. The relative velocity with respect to the Javelin was defined as the sum of the main inflow velocity and the velocity caused by the vibration. The main inflow against the javelin was assumed to be same over the longitudinal axis. However, the speed of main inflow with respect to the downstream side might be decreased in the wake, especially at lower *AoA*. As a result, the aerodynamic forces acting on the dynamic Javelin in the wake might be smaller when compared with the previous simulation.

It can be seen from Fig. 11 that the aerodynamic coefficients of a vibrating javelin are almost constant with respect to *AoA* around *AoA* of 0° even if *AoA* changes from time to time. It can be considered that the amplitude of *AoA* at 0° is very small, and in addition, the slopes of the aerodynamic coefficients with respect to *AoA* around 0° are gentle as shown in Fig. 10.

In future studies, the deformation of the Javelin should be measured simultaneously with the aerodynamic forces acting on the vibrating javelin. Moreover, a Bayesian optimization of the flight distance in consideration of the precise shape and stiffness of the Javelin in which the fluid–structure interaction problems are considered should be carried out.

## Conclusion

To accurately measure the aerodynamic forces acting on a Javelin without support interference, the world’s largest magnetic suspension and balance system was used. By utilizing a notch filter (band-stop filter for the resonance frequencies) and varying its intensity, both the static and dynamic (vibrating) cases were measured. It was found that the modern Javelin has positive values of the pitching moment coefficients (nose-up) at lower angles of attack. The pitching moment becomes positive, if the upstream side of the center of gravity gets more inflow than the downstream side. The time averaged aerodynamic forces of the dynamic case were comparable to those of the static case.

## Data Availability

The datasets used and analyzed during the current study available from the corresponding author on reasonable request.
